# Technological Devices and Their Effect on Preschool Children’s Eating Habits in Communities of Mixed Socioeconomic Status in Istanbul; a Pilot Cross-Sectional Study

**DOI:** 10.3390/bs11110157

**Published:** 2021-11-15

**Authors:** Aleksandra S. Kristo, Nur Çinar, Stefanos L. Kucuknil, Angelos K. Sikalidis

**Affiliations:** 1 Department of Food Science and Nutrition, California Polytechnic State University, 1 Grand Ave., San Luis Obispo, CA 93407, USA; asikalid@calpoly.edu; 2 Department of Nutrition and Dietetics, Istanbul Yeni Yuzyil University, Yilanli Ayazma Yolu No 26, Istanbul 34010, Turkey; nurcinar@yeniyuzyil.edu.tr (N.Ç.); stefankucuknil@yeniyuzyil.edu.tr (S.L.K.); 3 Balıklı Greek Hospital, Physiotherapy Clinic, Belgrad Kapi Yolu No 2, Istanbul 34020, Turkey

**Keywords:** preschool children, eating behaviors, technological devices, weight status

## Abstract

The use of technological devices is increasing in all age groups, especially in preschool-aged children. However, there is a limiting number of studies investigating the relationship between the use of technological devices, eating behavior, and weight status in preschool children. The aim of this study was (1) to describe total usage of technological devices, (2) to evaluate level of exclusive use of such devices by children, (3) to investigate children’s eating behaviors and diet in relation to screen time and type. A cross-sectional study was conducted with 104 children aged 2–5 years in Küçükçekmece and Bakırköy, Istanbul. Data collection consisted of a validated parental questionnaire on demographics and the child’s use of technology and eating behavior, while associations in children were examined using logistic regression analysis. The analysis of the obtained data uncovered a strong association between children’s TV and tablet/smart phone use and the foods consumed by children when using these devices (*p* = 0.0001; *p* = 0.012). Also, there was a significant association between children’s eating habits and TV, and tablet/smart phone durations of use (*p* = 0.015; *p* = 0.025), but not with computer duration of use (*p* > 0.05). Obesity and associated health problems can arise as results of suboptimal eating behavior, such as the ones observed in our study, which are also intensified with an increased duration of use of technological devices. The attitude of families towards prudent use of technological equipment is of great importance in impacting present and future health.

## 1. Introduction

In preschool ages, screen viewing constitutes one of the most common sedentary behaviors [1]. Screen viewing includes several aspects such as television, computers, smart phones, and tablets [2]. International statistics report that children in the United States (i.e., 4–7 years), in Canada (i.e., 3–4 years old), ref. [3,4,5] and Australia (i.e., 2–6 years) [6,7], as well as in India [8], are spending between 1.5 to 7.0 h per day screen viewing. Screen viewing is identified as a considerable risk factor for overweight and obesity [9,10,11]. Increased screen-time is positively associated with the development of obesity, which in turn increases the risk of several chronic diseases such as type 2 diabetes, fatty liver disease, as well as endocrine and orthopedic disorders [12,13].

More specifically, there is an association between TV viewing and the intake of high-caloric beverages and snacks [14,15,16]. Furthermore, children’s diet is affected by the advertising of energy-dense food on TV [17]. The combination of screen-time and exposure to TV advertising of foods is also shown to increase snacking and “junk food” consumption [18]. Also, the presence of TV in children’s rooms has been shown to increase TV-associated screen-time [19,20]. A series of Kaiser Family Foundation (KFF) studies reported that 4–27% of children younger than 6 years age used a computer for almost 1 h per day, while according to Christakis et al., daily use of television was 1.45 h and computer games 0.54 h in these age groups [21]. Additionally, research showed that boys tend to spend more time on computers than girls [22]. In that regard, the role of parents should be considered regarding usage of technology tools and children’s eating habits, given that eating habits are shaped at a young age [23] and parents play a significant role in regulating use and screen-time [24]. The American Academy of Pediatrics has suggested that viewing time should be limited to 1–2 h per day and that no TV or other screens should be allowed in children’s bedrooms [25]. In preschool children, the consideration is more critical in Australia as it is suggested that 2–5-year-old children should be limited to less than 1 h per day of screen-time and usage of other technological tools [26].

Although many studies show associations between TV viewing and the intake of high-calorific foods and drinks [27], there are limited data on the associations of other technologic devices and the foods and beverages consumed by children. Additionally, the advancement of technology, with the presence or frequency of tablet, smart phone, computer, and TV use in children between the ages of 2–5 years, is increasing rapidly [28]. Turkey is undergoing an epidemiological transition with Western practices and habits rapidly being adapted by a more traditional society facing increasingly more public health challenges, including high rates of childhood obesity, type 2 diabetes mellitus, and cardiovascular disease (CVD) [29,30]. Populations in Turkey are significantly understudied, thus more data are needed in this regard.

The objectives of this community-based study were to investigate the eating behavior and diet quality of Turkish preschoolers in relation to their screen habits, including time spent and type of device. We hypothesized that the use of technological devices in a sample of Turkish preschoolers would negatively affect their eating habits and weight status.

## 2. Materials and Methods

### 2.1. Sample

The study herein was a pilot cross-sectional study aiming to assess screen-time in preschoolers and the association with diet quality/habits of preschoolers from communities of mixed socioeconomic status in Istanbul by estimating the level, type, and number of technological devices used as well as diet quality/habits, in a sample of children between 2–5 years old fully enrolled to crèches (i.e.,: daycare-preschool). The questionnaire was applied in the following crèches all located in mixed socioeconomic status neighborhoods of Istanbul: “İkiz Kalpler Anaokulu”, “Gümüş Çocuklar Anaokulu” in Küçükçekmece, and the crèches which are found in Mazhar Osman Soul and Nervous Diseases Hospital in Bakırköy, in Istanbul. 27 questionnaires from “İkiz Kalpler Anaokulu”, 60 questionnaires from “Gümüş Çocuklar Anaokulu”, and 21 questionnaires from crèches found in Mazhar Osman Soul and Nervous Diseases Hospital, totaling 108 questionnaires, were obtained. Inclusion criteria for enrollment were as follows: children 2–5 years old, fully enrolled in crèches, without medical condition and/or on medication. A written informed consent form was obtained from the parents/legal guardians of enrolled children, while all the protocols and procedures were approved from the institutional IRB committee of Istanbul Yeni Yuzyil University (approval code: SBF-130705005), prior to the commencement of the study.

### 2.2. Tools—Questionnaire

Student participants’ parents in the study were provided the questionnaire in the presence of a trained registered dietitian and their children’s’ teacher. The questionnaire used was a validated one for the population administered to and was developed specifically to assess the association between TV viewing, computer use, and overweight, as well as determinants and competing activities of screen time in 4- to 13-year-old children [31,32]. The questionnaire asked parents to report the number of television sets, computers, tablets/smart phones, and game consoles, and the time that their child spends with these devices per day. The questions were multiple-choice and yes/no questions. Questions were designed to ascertain the rules for using these devices and if children had any device that was considered and/or was practically used by them only. Moreover, questions about food consumption and dietary habits/behavior during the use of devices were also included. Furthermore, parents’ demographic information was collected including age, educational status, and income, in addition to the child’s age, weight, and height. Partially completed questionnaires were not used and were excluded from the study. Out of 108 total participants enrolled in the study, there were 104 fully completed questionnaires that were used in the final analyses.

### 2.3. Statistical Analysis

The SPSS 20 Statistical Program statistical suite was used for data analysis. Significance was accepted at 0.05. Frequency, percentages, mean, and chi-square test analysis were used for demographics, use of technological devices, and eating behaviors. For other analyses, multinomial logistic regression analysis was used. Also, the children’s weight status was classified according to the USA’s Center for Disease Control and Prevention’s (CDC) data [33].

## 3. Results

### 3.1. Community Demographic Characteristics

The study’s population consisted of 104 pre-school children (age: 2–5 years) and their parents/legal guardians, more specifically 96 mothers, 5 fathers, and 3 grandmothers (as legal guardians). The age distribution of participating parents/legal guardians was 20.2% (*n* = 21) 21–30 years, 71.2% (*n* = 74) 31–40 years, 5.8% (*n* = 6) 41–50 years, 1% (*n* = 1) 51–60 years, and 1.9% (*n* = 2) in the 60+ age group. In terms of highest education level attained by mothers, 1% (*n* = 1) was illiterate, 1.9% (*n* = 2) primary school graduate, 4.8% (*n* = 5) middle school graduate, 40.4% (*n*= 42) high school graduate, 45.2% (*n* = 47) university graduate, and 6.7% (*n* = 7) postgraduate level. The corresponding statistics for the fathers were as follows: 1% (*n* = 1) of fathers were illiterate, 3.8% (*n* = 4) primary school graduate, 11.5% (*n* = 12) middle school graduate, 32.7% (*n* = 34) high school graduate, 42.3% (*n* = 44) university graduate, and 8.7% (*n* = 9) postgraduate level. In terms of gainful employment, 23.1% (*n* = 24) were not employed, while 76.9% (*n* = 80) of mothers worked full time jobs. All the fathers were working full time (Supplementary Materials: Table S1).

In terms of the sex and age group of the children-participants in this study, 39.4% (*n*= 41) of the children were girls and 60.6% (*n* = 63) boys. Age-wise 5.7% (*n* = 6) of children were 2 years old, 21.1% (*n* = 22) 3 years old, 42.3% (*n* = 44) 4 years old, and 30.8% (*n* = 32) 5 years old. The mean body weight of the girl participants was 16.1 ± 2.4 kg, while the mean of weight of boys was 17.8 ± 3.0 kg. Height for girls was 101.1 ± 9 cm on average, and for boys 104.5 ± 7.5 cm. Children’s weight status was calculated based on children’s body weight and length/height. Specifically, 14.4% (*n* = 15) of children were found to be underweight, 61.5% (*n* = 64) were of normal weight, 9.6% (*n* = 10) were overweight, and 14.4% (*n* = 15) were obese. Hence, overweight and obese combined, comprised 24% of study participants.

### 3.2. Screen Usage by Children

Regarding the television viewing rates of the children participants 44.2% (*n* = 46) of the children watch TV 0–1 h/day, 36.5% (*n* = 38) watch 1–2 h/day, and 14.4% (*n* = 15) watch 2+ h/day, whereas 4.8% (*n* = 5) of children do not watch television. When use of tablet/smart phone rates are examined, 48.1% (*n* = 50) use a tablet/smart phone 0–1 h/day, 23.1 % (*n* = 24) use 1–2 h/day, and 7.7 % (*n* = 8) use 2+ h/day tablet/smart phones, while 21.2% (*n* = 22) of children do not use tablet/smart phones. Regarding computer rates, 7.7% (*n* = 8) use a computer for 0–1 h/day, 1.9% (*n* = 2) for 1–2 h/day, and 1% (*n* = 1) for 2+ h/day, whereas 89.4% (*n* = 93) does not use a computer.

Considering technological devices in the household, 55.8% (*n* = 58) of families had 1 TV, 40.4% (*n* = 42) had 2 TVs, and 3.8% (*n* = 4) had 2+ TVs in the household. In terms of computer availability, 62.5% (*n* = 65) of families had 1 computer, 7.7% (*n* = 8) had 2 computers, and 1% (*n* = 1) had 3+ computers, whereas 28.8% (*n* = 30) families do not have a computer in their household. Moreover, 53.5% (*n* = 56) of families had 2 tablet/smart phones, 33.7% (*n* = 35) had 3 tablet/smart phones, and 12.5% (*n* = 13) had 3+ tablet/smart phones in household.

When identifying exclusive use (practically effective ownership) by children, 15.4% (*n* = 16) of children had a TV in their room, whereas 84.6% (*n* = 88) of children did not. Additionally, 3.8% (*n* = 4) of children had a computer, whereas 96.2% (*n* = 100) of children did not. Also, 20.2% (*n* = 21) of children had their own tablet/smart phone, as opposed to 79.8% (*n* = 83) which did not.

Along with the presence of technological devices in the household, we investigated the rules governing the use of the existing devices by children. Parents whose children use these technological devices completed this section of the questionnaire. When rules for watching TV were considered, 56.6% (*n* = 56) of parents ensured rules about when their children can watch TV, and 62.6% (*n* = 62) had established rules on how long their children can watch TV. However, 43.4% (*n* = 43) of parents did not have any rules/conditions regulating when their children can watch TV, and 37.4% (*n* = 37) of parents did not have any rules about duration of TV watching for their children. When potential rules for use of tablet/smart phones by children were accounted for, 76.8% (*n* = 63) implemented rules about time and duration of use for tablet/smart phone by their children. However, 23.2% (*n* = 19) of parents did not have any rules pertinent to the use of tablet/smart phone by their children. In terms of computer use, 70% (*n* = 7) of parents had rules established about when their children could use the computer, and 80% (*n* = 8) of parents had rules about the duration of use. However, no rules were employed by 30% (*n* = 3) of parents on timing and 20% (*n* = 2) of parents on the duration of use for children’s home computer(s).

### 3.3. Eating Behaviors of Children Relative to Screen Use

When evaluating the eating behaviors of children, it is reported that 84.6% (*n* = 88) of children always had breakfast, 8.7% (*n* = 9) of children often had breakfast, 3.8% (*n* = 4) of children sometimes had breakfast, 2.9% (*n* = 3) of children rarely had breakfast, and there was no child reported to never have breakfast (Table 1). In terms of number/frequency of meals per day, it was observed that 58.7% (*n* = 61) of children always consumed 3 or more meals per day, 24% (*n* = 25) of children consumed often, 14.4% (*n* = 15) of children sometimes, and 2.9% (*n* = 3) of children rarely consumed 3 or more meals in a day. When looking at the consumption of fresh fruit of children, it was seen that 51% (*n* = 53) of children always consumed, 30.8% (*n* = 32) consumed often, 15.4% (*n* = 16) sometimes, 1.9% (*n* = 2) rarely, and 1% (*n* = 1) of children never consumed fresh fruit. Regarding vegetable consumption, it was observed that 29.8% (*n* = 31) of children always consumed vegetables, 32.7% (*n* = 34) consumed often, 24% (*n* = 25) sometimes, 10.6% (*n* = 11) of children rarely consumed, and 2.9% (*n* = 3) of children never consumed vegetables. The following consumption of milk and dairy products such as cheese and yoghurt was observed: 67.3% (*n* = 70) of children always consumed, 22.1% (*n* = 23) consumed often, 4.8% (*n* = 5) consumed sometimes, 4.8% (*n* = 5) of children rarely consumed, and 1% (*n* = 1) of children never consumed milk and dairy products. Consumption of starchy products, such as bread, rice, and pasta in children was evaluated as follows, 45.2% (*n* = 47) always, 38.5% (*n* = 40) often, 14.4% (*n* = 15) sometimes, 1.9% (*n* = 2) rarely consumed, while there was no child who never consumed starchy products. Looking at the consumption of fast food, such as hamburgers and pizza, it was observed that 24% (*n* = 25) of children never consumed, 54.8% (*n* = 57) rarely consumed, 20.2% (*n* = 21) consumed sometimes, 1% (*n* = 1) consumed fast food often, while there were no children who always consumed fast food. Accounting for soft drink consumption, such as cola and soda pop, it was seen that 68.3% (*n* = 71) of children never consumed, 26.9% (*n* = 28) consumed rarely, 3.8% (*n* = 4) sometimes consumed, 1% (*n* = 1) of children often consumed soft drinks, and there was no child that always consumed soft drinks. Finally, when looking at the consumption of packaged food, such as crackers, chips, and chocolate, it was observed that 13.5% (*n* = 14) of children always consumed, 18.3% (*n* = 19) often consumed, 34.6% (*n* = 36) consumed sometimes, 29.8% (*n* = 31) consumed rarely, and 3.8% (*n* = 4) of children never consumed packaged foods.

On Table 2, where the mean eating behavior score of children is displayed, it is observed that both girls and boys always or often had breakfast, consumed three or more meals, and consumed fresh fruits, starchy products, milk, and dairy products. Moreover, children often or sometimes consumed vegetables, rarely or never consumed soft drinks, and sometimes or rarely consumed packaged food. When looking at the consumption of fast foods, it is observed that girls rarely or sometimes consumed and boys never or rarely consumed fast foods.

A scoring rubric was used to evaluate the quality of eating habits of children. A 1 to 5 points margin was assigned for each questions’ answer option. According to this rubric, it is determined as for “having breakfast”; “consumption of 3 or more than 3 meals per day”; “consumption of fresh fruits”; “consumption of vegetables”; “consumption of milk and dairy products”; “consumption of starchy products” of children; the “always” option is 5 points; “often” option is 4 points; “sometimes” option is 3 points; “rarely” option is 2 points; and “never” option is 1 point. As for the “consumption of fast food”, “consumption of soft drinks”, and “consumption of packaged food” of children, the “always” option is 1 point; “often” option is 2 points; “sometimes” option is 2 points; “rarely” option is 4 points; and “never” option is 5 points. According to this predetermined score, when the highest scores are collected for all the questions, the highest score possible is 45 and the lowest score is 9 for the children’s eating behaviors quality assessment. The produced score (between 9–45 points) is sub-categorized into 3 score group rages: “9–21” (low), “22–33” (medium), and “34–45” (high). The data analysis producing a score for each child participant revealed that there were no children in the “9–21” range group (low score). 6.7% of girls (*n* = 7), 16.3% of boys (*n* = 17), and a total of 23.1% (*n* = 24) of children are in the “22–33” points group (medium), while 32.7% (*n* = 34) of girls and 44.2% (*n* = 46) of boys are in the “34–54” points group (high) (Table 3).

According to our results regarding the use of technological devices by children, it is observed that 15.4% (*n* = 16) of girls, 16.3% (*n* = 17) of boys, and totally 31.7% (*n* = 33) of children watched TV during their meal, whereas, 24% (*n* = 24) of girls, 44.2% (*n* = 46) of boys, and totally 68.3% (*n* = 71) of children did not watch TV during their meal. Also, 12.5% (*n* = 13) of girls, 20.2% (*n* = 21) of boys, and totally 32.7% (*n* = 34) of children use a tablet/smart phone during their meal, whereas 26.9% (*n* = 28) of girls, 40.4% (*n* = 42) of boys, and totally 67.3% (*n* = 70) of children do not. When computer use is considered, 1% (*n* = 1) of girls, 1% (*n* = 1) of boys, and totally 1.9% (*n* = 2) of children use the computer, whereas 38.5% (*n* = 40) of girls, 59.6% (*n* = 62) of boys, and totally 98.1% (*n* = 102) do not use a computer during their meal.

Specifically, for soft drinks, it was observed that 9% (*n* = 7) of children rarely consumed, 1.3% (*n* = 1) of children sometimes consumed, and 89.7% (*n* = 70) of children did not consume soft drinks. Also, it was seen that, 3.8% (*n* = 3) of children always consumed packaged foods, 6.4% (*n* = 5) often consumed, 32.1% (*n* = 25) sometimes consumed, and 28.1% (*n* = 22) rarely consumed, whereas 29.5% (*n* = 23) of children did not consume packaged foods while watching TV. When looking at the consumption of fresh fruit when watching TV, 20.5% (*n* = 16) of children always consumed, 33.3% (*n* = 26) often consumed, 34.6% (*n* = 27) sometimes consumed, and 5.1% (*n* = 4) rarely consumed, whereas 6.4% (*n* = 5) of children did not consume fresh fruit when watching TV. Moreover, it was seen that 5.1% (*n* = 4) of children always consumed pastry, 5.1% (*n* = 4) often consumed, 44.9% (*n* = 35) sometimes consumed, and 15.4% (*n* = 12) rarely consumed pastry, whereas 29.5% (*n* = 23) of children did not consume pastry in front of the TV. When consumption of nuts or dried fruits is considered, 11.5% (*n* = 9) of children always consumed, 16.7% (*n* = 13) often consumed, 44.9% (*n* = 35) sometimes consumed, and 7.7% (*n* = 6) rarely consumed whereas 19.2% (*n* = 15) of children did not consume nuts or dried fruits in front of the TV.

Similarly, when looking into the food consumption in front of the tablet/smart phone by the children, for soft drinks, it was observed that 4.7% (*n* = 2) of children rarely consumed and 95.3% (*n* = 41) of children did not consume soft drinks. Also, we saw that 4.7% (*n* = 2) of children always consumed packaged foods, 4.7% (*n* = 2) often consumed, 34.9% (*n* = 15) sometimes consumed, and 39.5% (*n* = 17) rarely consumed, whereas 16.3% (*n* = 7) of children did not consume packaged foods when using smart devices. Regarding fresh fruit, 7% (*n* = 3) of children always consumed, 30.2% (*n* = 13) often consumed, 32.6% (*n* = 14) sometimes consumed, and 20.9% (*n* = 9) rarely consumed, whereas 9.3% (*n* = 4) of children did not consume fresh fruit in front of the tablet/smart phone. Additionally, we observed that 2.3% (*n* = 1) of children always consumed pastry, 7% (*n* = 3) often consumed, 44.2% (*n* = 19) sometimes consumed, and 20.9% (*n* = 9) rarely consumed pastry, whereas 25.6% (*n* = 11) of children did not consume pastry in front of the tablet/smart phone. For dried nuts or dried fruits, 4.7% (*n* = 2) of children always consumed, 26.6% (*n* = 11) often consumed, 32.6% (*n* = 14) sometimes consumed, 14% (*n* = 6) rarely consumed whereas, and 23.3% (*n* = 10) of children did not consume nuts or dried fruits in front of the tablet/smart phone.

When consumption of foods in front of a computer by children is considered, we observed that only 6 children consumed food when using a computer. When these children and the foods that they consumed were considered, we found that, for soft drinks, 66.7% (*n* = 4) did not consume any, and 33.3% (*n* = 2) of children rarely consumed packaged foods; 16.7% (*n* = 1) of children rarely consumed, 66.7% (*n* = 4) of children often consumed, and 16.7% (*n* = 1) of children always consumed fresh fruits; 50% (*n* = 3) of children sometimes consumed, 16.7% (*n* = 1) often consumed, 16.7% (*n* = 1) always consumed, and 16.7% (*n* = 1) of children did not consume pastry; 33.3% (*n* = 2) of children often consumed, 16.7% (*n* = 1) sometimes consumed, 33.3% (*n* = 2) rarely consumed, and 16.7% (*n* = 1) of children did not consume nuts or dried fruits when using a computer. Overall, the computer and food engagement was very low, possibly because computers are typically used to perform some sort of work or gaming, so eating and engaging in activity simultaneously would be challenging and impractical.

When evaluating the association between duration of technological devices use and weight status of children, the use of tablet/smart phone was found to have a higher effect on children’s weight status compared to TV and computer use, although this effect was not statistically significant (*p* > 0.05) (Table 4).

However, the association between the type of food consumed in front of technological devices and weight status of the children appears significant in certain cases. More specifically, there is a strong association between the weight status of children and the foods that are consumed in front of the TV (*p* = 0.000) (Table 5). Also, there is an association between the weight status of children and the foods that are consumed in front of the tablet/smart phone (*p* = 0.012). However, the foods that are consumed in front of the computer were not significantly associated with the weight status of the children (*p* > 0.05).

Finally, when evaluating the association between duration of technological devices use and nutrition score of children, there appears to be a significant association in some cases. While there was not any association between nutrition score of children and computer use (*p* > 0.05), tablet/smart phone use and TV viewing were significantly associated with the nutrition score of children (*p* = 0.015; *p* = 0.025) (Table 6).

Regarding TV viewing duration, it was observed that parental age was significantly associated with TV viewing duration (*p* = 0.000) (Table 7). Also, it was found that there was a strong association between TV viewing duration and TV location (TV found in child’s room) (*p* = 0.010). Besides, TV viewing duration was associated with the foods that children consumed in front of TV (*p* = 0.007). However, we did not find an association between TV number in household and child’s TV viewing duration (*p* > 0.05). When tablet/smart phone duration of use was taken into consideration, we saw that tablet/smart phone duration of use was significantly associated with the tablet/smart phone that children have (*p* = 0.004). Also, rules about when and how often children can use tablet/smart phone were strongly associated with the tablet/smart phone duration of use (*p* = 0.000).

## 4. Discussion

Childhood obesity, being one of the most serious global public-health challenges of the twenty-first century, has demonstrated an over tenfold increase over the past four decades [34,35,36]. In recent years, in Turkey, childhood obesity rates, along with public health concerns regarding metabolic disease risk, have increased considerably [37]. Specifically, in Turkey, 13.0% of the children in preschool age were found to be underweight, 14.6% were overweight and 5.9% were obese, and the incidence of overweightness and obesity was found to be higher in boys than girls [37]. In our study, the respective figures obtained were 14.4% underweight, 61.5% normal, 9.6% overweight, and 14.4% obese. The childhood obesity in the US is generally over 18% [38]. The importance of early-formed dietary habits has been clearly delineated for preschoolers [34]. Furthermore, the importance of parental guidance and behavior in this regard has also been shown to significantly influence the outcome of dietary behavior and health of children, especially during the first 1000 days [35]. Nutrition in adulthood is highly influenced by the eating habits gained during childhood, while parents, teachers, and the environment are playing an important role in the formation of pre-school eating habits [39]. Children’s food preferences are shaped, leading to either beneficial or detrimental dietary habits into adulthood [40,41]. Our study herein examined, in a Turkish population, which technological devices are used by preschool children and how often, as well as how often and what preschool children eat while using technological devices; we also assessed the effects of the above factors on children’s weight status.

In our study, children watched TV more than 1.5 h per day, similarly to what was reported by Mendoza et al. [42] and Twarog et al., with the latter group underlining that more than 2 h per day of TV viewing significantly increases the risk for obesity [43]. Factors influencing duration of TV viewing included parental age, presence of TV in child’s bedroom, and types of foods consumed while watching TV, independent of house rules and number of TV sets in the household. Interestingly, in our study, there was no relationship found between TV viewing duration and weight status, possibly because of a low number of participants. Nonetheless, the types of foods that children consumed during TV viewing were strongly associated with the children’s weight status. Research with preschoolers by the Kaiser Family Foundation reported that 52% of children in 2011 use mobile devices, which increased to 75% in 2013. In our study, 78.9% of preschool children were found to use a tablet/smart phone.

According to Common Sense Media, children younger than 8 years spent about 15 min in 2013 viewing tablets or smart phones, which increased to 48 min per day in 2017. We reported here that children spent over 1 h daily with a tablet/smart phone. With this timeframe, there was an association between tablet/smart phone use duration and the foods that children consume during their tablet/smartphone use. Also, it was determined that tablet/smart phone use duration was significantly associated children having a tablet/smartphone. Interestingly, there is no association between tablet/smart phone use duration and children’s weight status. However, it is noteworthy that while overall the dietary habits of children are not considered unhealthy, as determined by what they consume, it is interesting that the unhealthy choices are typically consumed during screen time. Similar results have been reported by studies in different settings, such as in the ALADINO study in Spain [44] and through the WHO European Childhood Obesity Surveillance Initiative [45]. Interestingly, in studies conducted in Australia, children from Middle Eastern cultural backgrounds demonstrated increased junk food consumption. More specifically, high junk food consumers were more likely to consume take-away ≥3/week, eat dinner in front of the television, receive sweet rewards, be allowed to consume snacks anytime, have soft drinks available at home, and have a TV in their bedroom [46].

In our study, we found that computer use among preschool children is very low, similar with previous studies [4]. When immature motor skills are considered, it is not surprising that the use of computer was low. Neither was surprising the lack of association between weight status and eating behaviors of children, for the same reason. While the data may not reflect a grave situation at this point, a variety of recent studies have indicated that increased screen time induces deviation from optimal dietary practices for children [47,48]. Moreover, Herman et al. demonstrated, through analyzing NHANES data, that there is a strong correlation between screen time and obesity that increases risk for future health issues [49]. Additionally, several studies have underlined the importance of parenting and rules’ establishment to improve outcomes associated with screen time, sedentary lifestyle, and quality of diet [1,31]. In our study, pertinent to the Turkish setting, we did see that there were cases where no rules were established regarding screen time, which is certainly alarming as a potential source of suboptimal habits and behavior setting as related to diet and health, with previous work having demonstrated how the quality of one’s dietary habits is associated with scholastic performance in Turkey [50]. While parental role can be pivotal in establishing good dietary habits regardless of socioeconomic status, in Turkey [51], it is important to work in a way to educate parents regarding this critical aspect of their role and how it can be best served. While there is no specific ideal diet, there are dietary paradigms such as that of the Mediterranean diet, appropriate to the Turkish setting, that have been shown to lower risk towards obesity and ensuing metabolic diseases both in adolescent and adults [52]. Finally, another component that needs also to be included in efforts and approaches targeting better health includes physical activity and exercise in combination with the nutritional changes. Exercise can contribute significantly to improved body composition and subsequent reduction of risk for chronic diseases independent of age [53].

The data collection tools used in this research are limited to the validated questionnaire used and the mode of selection which was semi-convenience. Our study also does not have information about what children watched when using technological devices. For this reason, it has not been possible to determine whether programs, advertisements, and applications are related to children’s weight status and eating behavior, although the literature clearly suggests that there is a strong relationship. To the best of our knowledge, this is the first study of its kind to report the relationship between technological devices use, eating behavior, and weight status in preschool children in Turkey.

## 5. Conclusions

In this study, we noted that the use of technological devices in preschool aged children was high, although most of the children’s weight status was within the normal range, while overweight and obesity combined was documented for 24% of our sample. Nevertheless, it has been observed that the food consumption increases based on the increased duration of use of these devices. Even if children are currently at a normal weight, obesity and other health problems may arise if these practices intensify and are implemented over extended periods of time. The attitude of families towards the prudent use of technological equipment is a determinant of their children’s present and future health.

## Figures and Tables

**Table 1:** Eating behavior patterns/frequency of children.

Eating Behaviors	Never		Rarely		Sometimes		Often		Always	
	*n*	%	*n*	%	*n*	%	*n*	%	*n*	%
Breakfast	0	0.0	3	2.9	4	3.8	9	8.7	88	84.6
3 or more meals daily	0	0.0	3	2.9	15	14.4	25	24.0	61	58.7
Fresh fruits	1	1.0	2	1.9	16	15.4	32	30.8	53	51.0
Vegetables	3	2.9	11	10.6	25	24.0	34	32.7	31	29.8
Milk and dairy products	1	1.0	5	4.8	5	4.8	23	22.1	70	67.3
Starchy foods/products	0	0.0	2	1.9	15	14.4	40	38.5	47	45.2
Fast food	25	24.0	57	54.8	21	20.2	1	1.0	0	0.0
Soft drinks	71	68.3	28	26.9	4	3.8	1	1.0	0	0.0
Packaged food	4	3.8	31	29.8	36	34.6	19	18.3	14	13.5

**Table 2:** Children Eating Behavior Score.

Eating Behavior	Girl (*n* = 41)	Boy (*n* = 63)	Total (Combined)
Breakfast	4.73 ± 0.74	4.76 ± 0.49	4.75 ± 0.51
3 or more meals daily	4.20 ± 0.44	4.51 ± 0.51	4.38 ± 0.44
Fresh fruits	4.37 ± 0.42	4.24 ± 0.46	4.29 ± 0.44
Vegetables	3.93 ± 0.41	3.65 ± 0.42	3.76 ± 0.44
Milk and dairy products	4.61 ± 0.49	4.43 ± 0.46	4.50 ± 0.44
Starchy foods/products	4.22 ± 0.45	4.30 ± 0.44	4.27 ± 0.45
Fast food	3.95 ± 0.42	4.06 ± 0.43	4.01 ± 0.43
Soft drinks	4.56 ± 0.47	4.65 ± 0.50	4.61 ± 0.48
Packaged food	2.95 ± 0.32	2.90 ± 0.31	2.92 ± 0.33

Values are means ± SEM. Total score refers to all participants combined (data not parsed by sex).

**Table 3:** Children’s Nutrition Score.

Nutrition Score	Girl		Boy		Total (Combined)	
	*n*	%	*n*	%	*n*	%
9–21 points	0	0.0	0	0.0	0	0.0
22–33 points	7	6.7	17	16.3	24	23.1
34–45 points	34	32.7	46	44.2	80	76.9

**Table 4:** Association between duration of technological devices use and weight status of children.

	Chi-Square	*p*-Value
TV viewing	6.585	0.680
Tablet/Smart phone use	13.733	0.132
Computer use	6.079	0.732

Statistical significance set at: *p* < 0.05.

**Table 5:** Association between foods consumed in front of technological devices and weight status of children.

	Chi-Square	*p*-Value
TV viewing	86.921	0.000
Tablet/Smart phone use	72.653	0.012
Computer use	12.137	0.276

Statistical significance set at: *p* < 0.05.

**Table 6:** Association between duration of technological devices use and nutrition score of children.

	Chi-Square	*p*-Value
TV viewing	71.775	0.015
Tablet/Smart phone use	69.015	0.025
Computer use	5.548	0.136

Statistical significance set at: *p* < 0.05.

**Table 7:** Association between TV viewing duration, and parental age, location of TV (child’s room) and types of foods.

	Chi-Square	*p*-Value
Parental age	84.893	0.000
Location of TV (child’s room)	73.465	0.010
Types of foods consumed	79.854	0.007

Statistical significance set at: *p* < 0.05.

## Data Availability

Data are available upon request and approval from the corresponding author Dr. Aleksandra S. Kristo.

## Supplementary Materials

The following is available online at https://www.mdpi.com/article/10.3390/bs11110157/s1, Table S1: Community Demographic Characteristics.
