# Supplementary material for: Technological Devices and Their Effect on Preschool Children’s Eating Habits in Communities of Mixed Socioeconomic Status in Istanbul; a Pilot Cross-Sectional Study
Source: Behav Sci (Basel). 2021 Nov 15;11(11):157. doi: 10.3390/bs11110157 (PMC8614987; doi:10.3390/bs11110157)
Supplement: Supplementary file 1 [file behavsci-11-00157-s001.zip › behavsci-1441356-supplementary.pdf]

Supplementary Material

Table S1. Community Demographic Characteristics.

**Demographics**	**n**	**%**
Parental/Guardian Role:		
Mother	96	92.3
Father	5	4.8
Grandmother/grandfather	3	2.9
Parent's Age (years):		
21-30	21	20.2
31-40	74	71.2
41-50	6	5.8
51-60	1	1.0
60+	2	1.9
Mother's Education level:		
Literate	1	1.0
Primary school	2	1.9
Middle school	5	4.8
High school	42	40.4
University	47	45.2
Postgraduate	7	6.7
Father's Education level:		
Literate	1	1.0
Primary school	4	3.8
Middle school	12	11.5
High school	34	32.7
University	44	42.3
Postgraduate	9	8.7
Mother employed		
Yes	80	76.9
No	24	23.1
Father employed		
Yes	104	100.0
No	0	0.0
